# Clinical Lectures on Diseases of the Eye

**Published:** 1866-02

**Authors:** E. L. Holmes

**Affiliations:** Lecturer on Diseases of the Eye and Ear in Rush Medical College, and Surgeon to the Chicago Charitable Eye and Ear Infirmary


*Clinical Lectures on Diseases of the Eye.* By E. L. HOLMES, M. D., Lecturer on Diseases of the Eye and Ear in Rush Medical College, and Surgeon to the Chicago Charitable Eye and Ear Infirmary.

### MYOPIA—SHORTSIGHTEDNESS.

GENTLEMEN,—The condition of the eye in those cases, in which distant objects can only be distinctly seen with the aid of concave lenses, has not till comparatively recently been fully analyzed and comprehended. The term myopia has generally been applied to *all* these cases, which, however, are now in strict diagnosis separated into two clearly distinct classes. Unfortunately the words, which might distinguish these two classes, are synonymous in our language, and cannot be well applied to them. Further explanation will show you the difference in the classes to which the English terms, apparently without distinction, have been given. These are shortsightedness, or Brachymetropia, and nearsightedness, or Plesiopia.

As there is an infinite variety of cases in each class as regards *degree*, and as there is a point in a series of cases from the two classes, where a difference could scarcely be detected, it should be borne in mind, that I shall only speak of typical cases, such as I have occasionally shown you, although we have not fully examined their peculiarities.

In the first class of cases, there is an abnormal formation of the globe itself, the antero-posterior diameter of the eye being too long. This elongation of the eye may be dependent upon a congenital tendency to its development, or may be acquired. It is worthy of notice that in infancy, the crystalline lens possesses a form so nearly spherical, and is placed so near the cornea, that the eye is myopic. As the infant develops, the lens becomes less convex and recedes from the cornea, and the eye in normal cases is able by the power of adjustment to see distant as well as near objects.

Sometimes, however, the eye does not develop normally, but in such a way that the antero-posterior diameter tends to increase more rapidly than the other diameter, consequently the retina is at so great a distance behind the lens, that the eye becomes permanently myopic. The greatest relaxation possible of the apparatus of adjustment cannot sufficiently diminish the convexity of the lens, to enable rays of light from distant objects to come to a focus upon the retina. The focus for distant objects in such a condition of the eye, is *in front* of the retina, near objects alone by the greater divergency of their rays can form distant images on the retina. The inability to see distant objects is sometimes further increased by too great convexity of the crystalline lens.

Myopia may be so slight in degree that the patient is scarcely aware of any defect, or it may be so great that ordinary print can only be seen at the distance of two inches, or even of an inch and a half; such patients scarcely see sufficiently well to guide themselves safely in the street.

The causes of this elongation of the globe are obscure; they do not appear to depend upon any recognized diathesis; it is said to be often hereditary and dependent upon abnormal fœtal development; it is liable to increase with age, especially where it is already quite excessive in childhood. The presence of a slight elongation cannot be observed by simple inspection. In marked cases it can be readily seen by the projection of the anterior half of the globe, especially when the eye is rotated. The cornea, except in marked cases, retains its normal convexity, the apparent change in its form being caused by the great distance between the iris and cornea.

The ophthalmoscope reveals in a large number of these cases a peculiar yellow appearance in the form of a cone around the temporal side of the papilla of the optic nerve. This cone is well represented in the plates before you, and was seen by a few of you in the case of the young lady, recently examined with the ophthalmoscope. This peculiar conical figure is produced by an attenuation of the choroid, near the optic papilla. As the sclerotic is also attenuated and consequently weakened, it fails to give sufficient support for the normal pressure outward of the intraocular fluids; hence, we have a projection posteriorly called staphyloma posticum.

At one time this condition was supposed to depend upon inflammation. As none of the ordinary symptoms of inflammation during life, or its products after death are observed, and as the retina usually retains its delicacy of perception, this opinion is now generally abandoned. It is undoubtedly, as has already been said, due to abnormal development sometimes noticed at the time of birth, and liable to increase during youth, and sometimes even later in life. Some have believed that it was produced by undue use of the eye upon near objects. The fact that it is often congenital, and that it is found as often among the classes of people whose occupation does not require the use of the eyes upon near objects, as among those who do thus employ their sight, shows that such an opinion is incorrect.

Vision is generally within certain limits remarkably good in ordinary cases of myopia. The eyes can be used for a longer time upon minute objects, and with less light than the normal eye; the internal recti muscles from constant use in very many cases become more developed, and are able to retain the visual axes of the eyes convergent more easily than those of the normal eye. Although in many instances, shortsightedness may be arrested in its progress, it not unfrequently increases in degree. As the globe elongates, the choroid and retina, finally present symptoms of congestion and inflammation, of which amaurosis may be the result.

Shortsightedness is sometimes *acquired*, although not so frequently as was formerly supposed. Since so many persons, engravers and jewelers for instance, constantly employ their sight upon near and fine objects, without acquiring shortsightedness, it may be supposed that in cases where it is acquired, there is a peculiar predisposition to it.

Certain forms of choroidal inflammation, general congestion of the eyes from their excessive use on minute objects with insufficient light, increase in the degree of convexity of the cornea from disease, all may tend to increase the length of the anterio-posterior diameter of the globe, thus rendering the eye myopic.

It is a popular opinion that individuals, who are shortsighted in youth, usually have normal vision in old age. This opinion is erroneous, as regards the great majority of patients with elongation of the globe. We shall soon see in what class of cases age tends to render the eyes less "nearsighted."

We have thus seen that shortsightedness—Brachymetropia—is a malformation of the eye—a disease, which is liable to progress and end in more or less discomfort, and even amaurosis. The apparatus of accommodation may remain unimpaired, the increased distance of the retina from the cornea counteracting the utmost efforts of this apparatus to reduce the convexity of the lens to such a degree, that rays of light from distant objects (parallel or nearly parallel rays) can be brought to a focus so far behind the lens.

Nearsightedness, or Plesiopia, is that condition of the eye, in which the form is normal as regards its diameter, but in which there is simply a reduction of the power of accommodation for distant objects. While the contraction of the ciliary muscle so increases the degree of convexity, that the eye is accommodated for near objects, not nearer, however, than five inches, the relaxation of the ciliary muscle, and the consequent tension of the Zonula of Zinn, cannot flatten the crystalline lens to such an extent, that rays from distant objects can form a focus on the retina. This condition of the eye is often produced in childhood and youth, when from the delicacy of the structure of the lens, the constant use of the eyes upon near objects, retains the lens habitually in so great a degree of convexity, that, as its substance becomes firmer with the increase of age, it remains in a more convex form, and the adjusting apparatus cannot produce flattening of the lens to its normal extent.

It is in this form of myopia, that the eye becomes with advanced age normal as regards seeing distant objects, or even *farsighted*—and for the reason, that advanced age is usually attended with absorption of the fluid portions of the lens, producing a flattening of this organ. Hence in cases, where in youth there may be too great convexity in advanced age, the absorption of the fluids of the lens may give a degree of flattening, adapted to vision of distant objects.

The treatment in ordinary cases of both these forms of myopia is simple, and is almost wholly confined to the use of concave lenses and suitable use of the eyes with proper exercise in looking at distant objects.

Where there is a tendency to any increase of myopia—Brachymetropia—great care should be observed on the part of the patient in the use of the eyes, and of the physician in the examination of the case and in the treatment. All such patients, especially children, should be taught not to bring objects too near the eyes, and to avoid bending the head forward in reading or writing. They should do nothing which tends to produce congestion of the globe. Consequently, long continued efforts on the part of the internal recti and ciliary muscles, as in reading fine print, should be avoided. Strict attention to the condition of the abdominal organs should be observed, in order to prevent secondary congestions of the head. Of course, great care should be taken in regard to the selection of an occupation.

A few words only are here necessary regarding the use of concave lenses. As you all know, these lenses render rays of light radiating from objects more divergent, the degree of increase of divergency depending upon the degree of concavity of the lens. The increase in the degree of divergency with which the rays fall upon the convex surface of the cornea, causes them to come to a focus further behind the lens.

Although it requires very delicate and expensive optical instruments to measure the exact distance of objects from the cornea, for which the eye can be adjusted, and consequently to calculate the precise degree of concavity required in a lens suited to each case of myopia, for all practical purposes in nearly all cases, the following rule is sufficiently accurate:

If from the greatest distance, at which the patient can distinctly read quite fine print measured in inches, one half an inch, (the distance from the cornea at which lenses are usually worn) be subtracted, the remaining number will give the focal distance of the required lens.

